# Lifecycle Management as a Roadmap to the Tobacco Endgame

**DOI:** 10.31586/wjcmr.2025.6181

**Published:** 2025-09-14

**Authors:** Shervin Assari, John Ashley Pallera, Mahmoud Efatmaneshnik

**Affiliations:** 1 Charles R Drew University of Medicine and Science (CDU), Los Angeles, CA, USA; 2 University of South Australia (UniSA), Mawson Lakes, Adelaide, Australia

**Keywords:** Tobacco Control, Lifecycle Management, Enterprise Architecture, Zachman Framework, Public Health Policy, Complex Adaptive Systems, Systems Thinking, Policy Design, Implementation, Evaluation, Endgame Strategies, United States, Health Governance, Regulatory Frameworks, Cross-Sector Alignment, Sustainability, Policy Coherence, Risk Mitigation, Industry Countermeasures, Public Health Systems

## Abstract

**Background:**

Tobacco endgame, defined as elimination of commercial tobacco sales The U.S. tobacco control landscape is a complex, adaptive system shaped by diverse stakeholders, evolving products and regulations, shifting social norms, and the strategic countermeasures of a powerful industry. Managing such complexity requires more than isolated interventions—it demands a coordinated, enterprise-wide approach that accounts for dynamic interactions, feedback loops, and emergent risks.

**Objective:**

Drawing on complex systems thinking, Zachman enterprise architecture model, and public health best practices, we conceptualize tobacco control as an evolving enterprise progressing through six interconnected phases: (1) Conception & Initiation, (2) Policy & System Design, (3) Implementation & Operation, (4) Evaluation & Adaptation, (5) Consolidation & Endgame Transition, and (6) Sustainment or Sunset. Each phase incorporates governance structures, performance benchmarks, and transition criteria designed to manage interdependence and reduce systemic vulnerabilities.

**Results:**

The lifecycle framing emphasizes how tobacco control in the U.S. can evolve as a complex, adaptive enterprise—integrating public health objectives with legal, operational, and cultural change processes. This model supports strategic sequencing, cross-sector alignment, and risk mitigation against emergent industry tactics, enabling a resilient and measurable pathway to the endgame.

**Conclusions:**

Seeing tobacco control as a complex enterprise that operates under a lifecycle model may offer a roadmap for achieving and sustaining the tobacco endgame. Using this approach may enhance policy coherence, resource efficiency, and adaptability, ensuring tobacco endgame is achieved.

## 1. Background

### 1.1. Introduction

Tobacco control operates within a highly complex, adaptive system that spans multiple levels of influence—ranging from cultural norms and product innovation to market forces and the overlapping layers of local, state, and national policy. These elements interact dynamically, shaped by diverse stakeholders, shifting social contexts, and the strategic countermeasures of a well-resourced tobacco industry. The result is a constantly evolving landscape in which interventions at one level can trigger cascading effects across others.

In systems science, complexity is not just an abstract descriptor; it is composed of identifiable, influential elements that can be measured, monitored, and managed [1]. These include the number and diversity of interacting components, the strength and direction of interdependence, the presence of feedback loops, and the degree of uncertainty or adaptability within the system [2]. Understanding and addressing these complexity dimensions is essential for designing robust, sustainable tobacco control strategies.

Despite the inherently complex nature of the tobacco control system, most U.S. efforts to date have addressed its components in isolation. Policies, programs, and advocacy campaigns have often been implemented reactively—responding to emerging threats such as novel nicotine products [3–9] —rather than through a coordinated framework that explicitly measures and manages complexity. This reactive approach has produced significant public health gains but also left gaps in resilience, equity, and long-term sustainability [10–16].

One way to systematically address and manage this complexity is to treat tobacco control as an enterprise—an organized, goal-directed system encompassing diverse actors, processes, and resources working toward a shared mission [17]. The Zachman Architecture Framework (ZAF) [18], originally developed to manage the complexity of large-scale information systems, offers a structured method for doing so. By mapping six fundamental interrogatives—What, How, Where, Who, When, and Why [19]—across different stakeholder perspectives and lifecycle phases, ZAF creates a matrix of “viewpoints” that break the system into manageable, measurable components [20]. This structured layering and abstraction make it possible to capture the multi-dimensional nature of tobacco control, identify gaps, and align actions across sectors [21].

Integrating a lifecycle perspective into this enterprise framework further enhances its utility. By conceptualizing tobacco control as progressing through predictable, interdependent stages—from conception and policy design to implementation, adaptation, endgame transition, and sustainment—policymakers can anticipate complexity before it becomes a barrier, sequence strategies strategically, and preserve gains over time. In this way, enterprise architecture, and ZAF in particular, provide a practical and evidence-based means of navigating the complexity that defines the tobacco control challenge [22,23].

### 1.2. Complexity of Tobacco Control

**Figure 1.**
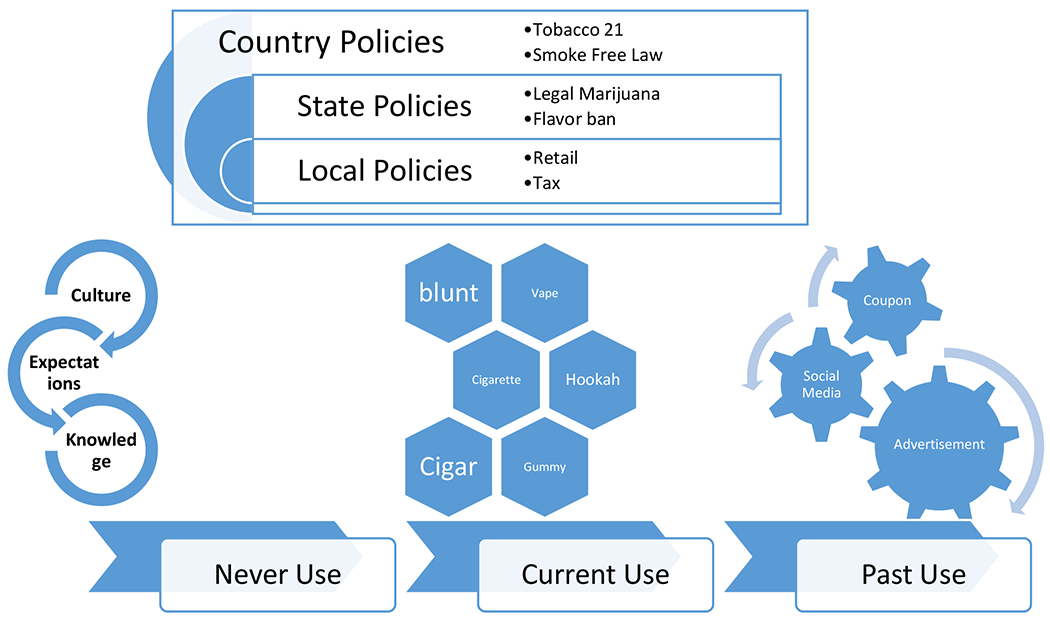
Some Elements of Complexity of Tobacco Control

As shown by Figure 1, tobacco use and its control are inherently complex, functioning much like a layered onion in which each layer represents interconnected influences and governance structures. At the innermost layer lies individual behavior, shaped by age, culture, personal beliefs, and access to products. Surrounding this are market forces, including the diversity of tobacco and nicotine products, industry marketing strategies, and socioeconomic factors that affect both exposure and vulnerability. Beyond these, multiple layers of policy and regulation—local, state, and national—interact to shape the environment in which tobacco use occurs. Local governments may impose retail restrictions or zoning laws, states may enact flavor bans or tax policies, and the federal government sets overarching standards such as age restrictions and product regulations. These policy layers are not static; they are dynamic systems influenced by political priorities, public opinion, health advocacy, and the counterstrategies of the tobacco industry. Together, these interdependent layers form a shifting policy ecosystem where changes at one level can ripple through others, underscoring the need for coordinated, multi-level approaches to achieve the tobacco endgame.

### 1.3. Lifecycle Framework for the Tobacco Control Enterprise

**Figure 2.**
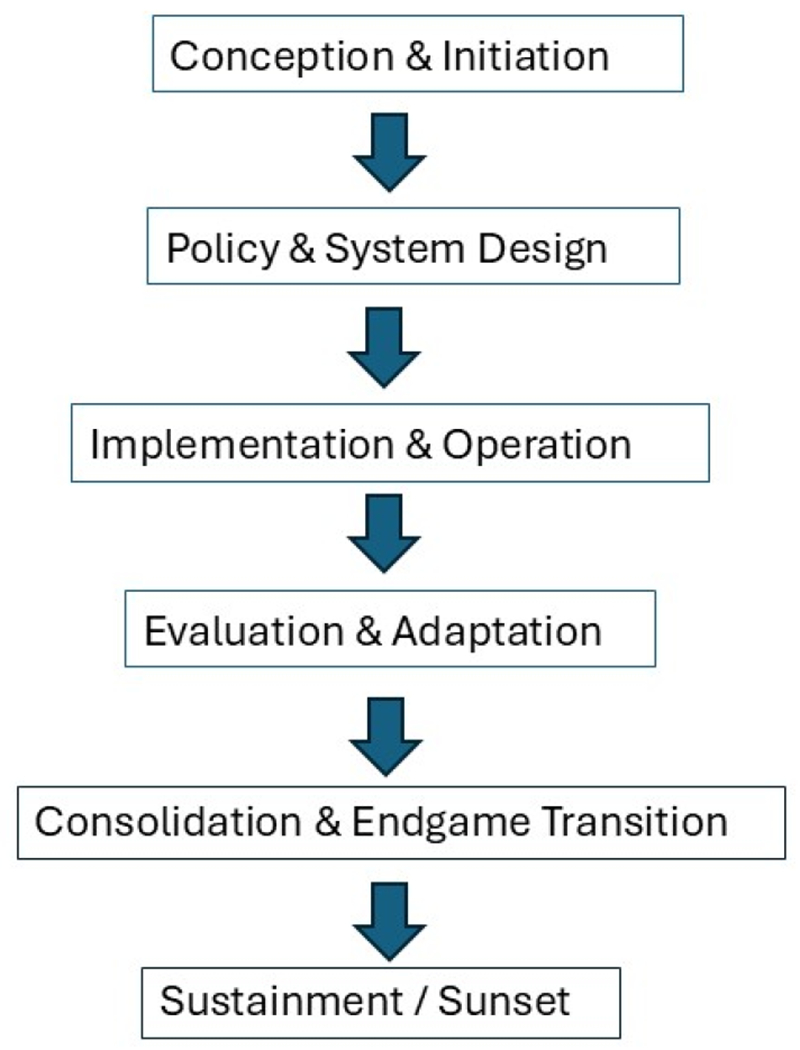
Lifecycle diagram

Figure 2 illustrates a six-phase lifecycle framework designed to guide the U.S. tobacco control enterprise from problem recognition to sustaining a post-tobacco environment. It begins with *Conception & Initiation*, where public health agencies identify the scale of the tobacco problem, gather evidence, and build political and community support. In *Policy & System Design*, these insights translate into laws, regulations, and operational plans aligned with national and international guidelines. *Implementation & Operation* then activates programs, campaigns, cessation services, and enforcement mechanisms to reduce tobacco use. The *Evaluation & Adaptation* phase ensures that policies remain effective and responsive to emerging challenges such as new nicotine products or illicit trade. As prevalence declines, *Consolidation & Endgame Transition* focuses on final, intensive measures like nicotine reduction, supply limits, and smokefree generation laws to eliminate commercial tobacco. Finally, *Sustainment / Sunset* maintains vigilance through long-term monitoring, community engagement, and education to preserve tobacco-free norms and prevent relapse. This structured progression ensures a comprehensive, data-driven, and adaptive approach to achieving the tobacco endgame.

### 1.4. Aims

This paper presents the Tobacco Control Enterprise Lifecycle Model, applying the Zachman Architecture Framework to integrate organizational lifecycle theory with global tobacco endgame strategies. The model is designed as a complexity management tool, structuring the many interdependent elements of tobacco control—data, processes, stakeholders, locations, timeframes, and motivations—into a coherent, phased architecture.

The primary aim is to provide a roadmap that enables U.S. tobacco control stakeholders to:

- Align diverse actors and functions within a shared enterprise architecture;
- Sequence actions strategically across six interconnected lifecycle phases;
- Embed adaptability to respond to emergent threats, shifting social norms, and technological changes in nicotine delivery;
- Assess and improve process maturity at each phase using the Capability Maturity Model Integration (CMMI) framework, ensuring that interventions evolve from ad hoc initiatives to optimized, continuously improving systems;
- Manage systemic complexity by mapping interdependencies, clarifying responsibilities, and preserving coherence across all levels of decision-making.

By combining complexity management principles with process maturity assessment, the Tobacco Control Enterprise Lifecycle Model reframes tobacco control from a reactive set of disconnected measures into a proactive, strategic, and sustainable national undertaking—capable of achieving and maintaining the tobacco endgame in the face of evolving challenges.

## 2. Methods

### 2.1. General

The development of the Tobacco Control Enterprise Lifecycle Model was informed by a synthesis of organizational lifecycle theory, complexity management principles, public health program planning frameworks, and historical analysis of tobacco control policy evolution in the U.S. and globally.

Organizational lifecycle theory, widely applied in business, engineering, and governance contexts, conceptualizes complex systems as progressing through predictable stages—from formation to maturity, adaptation, and eventual dissolution or transformation [24,25]. This theoretical lens was combined with the Zachman Architecture Framework (ZAF) to systematically structure the multiple dimensions of the tobacco control enterprise—What (data/elements), How (processes), Where (locations/networks), Who (stakeholders), When (timing), and Why (goals/motivations)—across six stakeholder perspectives. This structured mapping allowed for explicit identification of interdependencies, feedback loops, and potential points of systemic fragility.

Our approach involved the following steps:

1. Literature and Policy Review – We reviewed national and state-level tobacco control strategies, CDC Best Practices for Comprehensive Tobacco Control Programs [26], WHO FCTC guidelines [27,28], and peer-reviewed literature on tobacco endgame strategies. This established the evidence base for defining lifecycle phases and identifying stage-specific challenges.
2. Complexity Mapping with ZAF – We applied the Zachman Framework to map each lifecycle phase against its core interrogatives, creating a multi-dimensional architecture of the tobacco control enterprise. This provided a consistent framework for identifying missing elements, potential duplication, and weak interconnections.
3. Lifecycle Phase Definition – Based on both historical precedents (e.g., early smoke-free laws in California) and forward-looking endgame strategies (e.g., nicotine reduction, supply quotas), we defined six interconnected lifecycle phases: Conception & Initiation, Policy & System Design, Implementation & Operation, Evaluation & Adaptation, Consolidation & Endgame Transition, and Sustainment or Sunset. Each phase was described in terms of its primary purpose, key drivers, essential activities, intended outputs, and transition criteria to the next stage.
4. Process Maturity Assessment Framework – To assess and enhance the effectiveness of each phase, we adapted the Capability Maturity Model Integration (CMMI) [29] to the public health policy context. Each phase was assigned maturity level descriptors (Levels 1–5: Initial, Managed, Defined, Quantitatively Managed, Optimizing), enabling stakeholders to evaluate whether processes were ad hoc, repeatable, standardized, data-driven, or continuously improving.
5. Expert Consultation – We sought feedback from public health practitioners, policy advocates, and academic researchers to validate the relevance of the model, the appropriateness of maturity assessments, and the feasibility of implementing complexity mapping in operational settings.

Our approach involved mapping six distinct but interconnected phases: Conception & Initiation, Policy & System Design, Implementation & Operation, Evaluation & Adaptation, Consolidation & Endgame Transition, and Sustainment or Sunset. Each phase was defined by its primary purpose, key drivers, essential activities, and intended outputs. The sequence reflects both historical precedents in U.S. tobacco control—such as the early adoption of smoke-free laws in California [30,31]—and forward-looking strategies such as supply reduction measures.

Data sources for defining these phases included CDC’s *Best Practices for Comprehensive Tobacco Control Programs* [26], WHO FCTC guidelines [27,28], state and local tobacco control plans, and peer-reviewed literature on endgame strategies. Expert feedback was solicited from public health practitioners, policy advocates, and academic researchers to validate the model’s relevance and ensure alignment with current policy contexts. This qualitative validation helped refine the operational definitions of each phase and clarify the transitions between them.

### 2.2. Tobacco Control Enterprise Lifecycle Elements and CMMI Process Maturity Context

Table 1 presents a structured view of the U.S. tobacco control enterprise across six lifecycle phases, aligning each with the Zachman interrogatives—What (data and products), How (processes), Where (geographic scope), Who (stakeholders), When (timeframes), and Why (goals). This framework captures the tangible artifacts, operational functions, actors, and strategic intent at each stage, from initial problem recognition to long-term sustainment.

At the Conception & Initiation phase, the enterprise is driven by epidemiological evidence, mortality data, economic burden studies, and mission-setting documents, supported by processes like situational analysis and stakeholder mapping. This stage operates mainly in federal and state policy arenas and is focused on building political momentum—placing it around CMMI Level 2 (Managed), where processes are planned and documented but not yet fully standardized.

In Policy & System Design, tangible outputs such as draft legislation, regulatory frameworks, and performance metrics are developed through legislative drafting, regulatory impact assessment, and organizational design. Conducted in legislative chambers and government agencies, this phase requires cross-sector participation and aims for enforceable, evidence-based laws—aligning with CMMI Level 3 (Defined), where processes are standardized and integrated.

Implementation & Operation produces and mobilizes public campaign materials, cessation services, inspection regimes, and compliance systems. Execution occurs in community, educational, retail, and enforcement settings, with the goal of reducing initiation, boosting cessation, and ensuring compliance. Here, the maturity transitions toward Level 3–4, with standardized delivery enhanced by quantitative performance monitoring.

The Evaluation & Adaptation phase focuses on surveillance datasets, program evaluations, and emerging threat detection (e.g., illicit trade, vaping trends) . Processes emphasize data-driven policy review and stakeholder feedback integration, operating at CMMI Level 4 (Quantitatively Managed)—measuring and controlling operations based on robust performance metrics.

In Consolidation & Endgame Transition, the focus shifts to high-impact measures such as nicotine content reduction, supply “sinking lids,” and smokefree generation laws, supported by cross-border enforcement agreements. Coordination across national and international networks moves the system toward Level 4–5, incorporating continuous improvement and innovation to achieve the endgame goal of eliminating commercial tobacco.

Finally, Sustainment / Sunset ensures long-term prevention of relapse through ongoing monitoring, cultural reinforcement, and community-led education. With an emphasis on adaptive, proactive stewardship in a post-tobacco environment, this phase exemplifies CMMI Level 5 (Optimizing)—a system in perpetual refinement, using feedback to maintain a tobacco-free society.

### 2.3. Applying CMMI Levels to Public Health and Tobacco Control Policy

Table 2 adapts the Capability Maturity Model Integration (CMMI) framework—originally developed for process improvement in software and systems engineering—to the public health and tobacco control policy domain. The five CMMI levels provide a structured lens for assessing the maturity of tobacco control processes, from reactive, ad hoc interventions to a fully optimized, continuously improving system.

At Level 1 (Initial), tobacco control efforts are sporadic and highly dependent on individual initiatives, such as isolated pilot programs or local campaigns without coordinated enforcement. Moving to Level 2 (Managed), activities become more structured: projects are formally planned, documented, and tracked, with defined ownership and repeatable procedures at the program level. Level 3 (Defined) marks the point where processes are standardized and integrated across the national system, resulting in consistent policy frameworks and interagency coordination.

Level 4 (Quantitatively Managed) elevates this integration by embedding robust measurement and surveillance into decision-making. Here, performance metrics, prevalence data, and enforcement statistics actively shape program priorities and resource allocation. Finally, Level 5 (Optimizing) represents a mature system that uses continuous, data-driven feedback to adapt policies, anticipate threats from emerging products, and innovate in pursuit of endgame goals.

Framing tobacco control within this CMMI progression enables policymakers and stakeholders to identify their current stage of maturity, pinpoint capability gaps, and chart a path toward a fully optimized, adaptive system capable of sustaining tobacco endgame momentum.

### 2.4. Measures & Variables Across Zachman Cells for the U.S. Tobacco Endgame

Table 3 translates the Zachman Framework’s six interrogatives—What, How, Where, Who, When, and Why—into a structured set of measurable indicators for tracking and guiding progress toward the U.S. tobacco endgame. Each interrogative serves a distinct evaluative purpose and is applied consistently across all six lifecycle phases, from Conception & Initiation to Sustainment / Sunset, ensuring that strategy, execution, and adaptation are grounded in evidence and measurable outcomes.

For What (Data, Elements, Products), the focus is on the completeness and quality of the tangible artifacts that underpin the endgame effort. Early phases emphasize foundational assets such as baseline prevalence datasets and policy concept papers, while later phases track concrete outputs like legislated endgame measures, operational monitoring systems, and the dissemination of norm reinforcement materials.

How (Processes, Functions) assesses the maturity, consistency, and rigor of implementation. Initial phases measure the inclusivity and quality of stakeholder engagement and adherence to impact assessment standards, while operational phases monitor campaign effectiveness, compliance inspections, and the continuity of surveillance and educational programs into the post-endgame period.

Where (Locations, Networks) captures the breadth of geographic coverage and the integration of networks at each stage. This includes the proportion of states with reliable baseline data, alignment between state and federal policy frameworks, geographic reach of campaigns and enforcement, and post-endgame monitoring coverage in high-risk zones.

Who (Stakeholders, Actors) evaluates the diversity, representation, and coordination of the actors involved. Measures range from counting stakeholder categories engaged in the early stages to quantifying cross-agency coordination effectiveness during the transition phase, and community engagement in sustaining tobacco-free norms during the sunset period.

When (Timeframes, Events) examines timeliness and synchronization. Key indicators include the speed from problem identification to initial strategy drafting, policy drafting cycle times, on-time delivery of campaigns, rapid policy responses to emerging threats, and the national synchronization of final endgame measures.

Finally, Why (Goals, Motivations) ensures that objectives remain clear, measurable, and adaptable. From early mission statement specificity to explicit alignment with national or international health targets, and from mapping program KPIs to strategic goals to maintaining relapse prevention objectives post-endgame, this dimension keeps the initiative’s vision aligned with measurable public health outcomes.

Taken together, the measures and variables in Table 3 provide a multi-dimensional performance monitoring architecture. This approach not only facilitates accountability but also supports adaptive management by enabling policymakers to diagnose weaknesses, replicate successes, and sustain momentum across the entire lifecycle of the tobacco endgame.

### 2.5. U.S. Tobacco Endgame Zachman Cell Measures

Table 4 outlines a comprehensive measurement framework for guiding, monitoring, and evaluating the U.S. tobacco endgame effort across the full lifecycle of the tobacco control enterprise. Using the Zachman Framework’s interrogatives—What, How, Where, Who, When, and Why—the table operationalizes each lifecycle phase with measurable indicators, corresponding variables, and example data sources, including both traditional surveillance systems and potential AI-enabled search methods. This structure ensures that each phase, from initial conception to post-endgame sustainment, has clear, evidence-informed metrics for progress tracking and accountability.

The Conception & Initiation phase emphasizes baseline situational awareness and early coalition-building, highlighting indicators such as prevalence data availability, stakeholder diversity, and the specificity of mission statements. Policy & System Design focuses on legislative completeness, regulatory alignment, and multi-sector representation, drawing on resources like CDC STATE System datasets and legislative tracking tools. Implementation & Operation shifts toward execution metrics, such as campaign reach, inspection coverage, and adherence to program timelines, supported by operational data from Quitline reports, FDA compliance databases, and state performance plans.

The Evaluation & Adaptation phase centers on responsiveness, including the timeliness of surveillance datasets, speed of policy adjustments to emerging threats, and incorporation of evaluation findings into revised goals. Consolidation & Endgame Transition measures the enactment and synchronization of final supply-reduction policies, cross-agency coordination, and targeted cessation outreach, ensuring alignment with national prevalence reduction targets. Finally, Sustainment / Sunset addresses the long-term maintenance of tobacco-free norms through ongoing illicit trade monitoring, continued public health education, and community engagement, with attention to relapse prevention objectives.

By mapping each lifecycle phase to specific, measurable indicators and credible data sources, this framework not only supports systematic performance evaluation but also facilitates adaptive management. The explicit link between measures, variables, and data sources allows decision-makers to quickly identify gaps, track progress toward strategic objectives, and ensure that the tobacco endgame remains both evidence-based and outcome-oriented.

## 3. Results

### 3.1. General

Our review of the tobacco control landscape underscores the value of applying a lifecycle framework to understand how diverse actors, policies, and communication channels interact over time. Tobacco control in the United States is not the product of a single intervention but rather a dynamic enterprise involving global agreements, national legislation, state and local ordinances, corporate practices, activist campaigns, and media outreach. The findings presented in Tables 5 and 6 highlight how these activities span the full range of lifecycle phases—from early framing and policy design through implementation, evaluation, consolidation, and long-term sustainment. By drawing on internet-based resources, organizational websites, and social media communications, we captured not only the formal policy infrastructure but also the parallel role of advocacy groups, schools, health systems, researchers, and even the tobacco industry in shaping trajectories. Taken together, these results illustrate how tobacco control evolves as a complex, adaptive system, and how real-world examples across multiple levels and sectors can inform strategies for achieving and maintaining the endgame.

### 3.2. Six-Phase Lifecycle: Real-World Examples

Table 5 provides a summary of real-world examples aligned with the six-phase lifecycle framework. These examples, identified through our targeted search of internet-based resources, illustrate how tobacco control has unfolded across global, national, state, and local contexts. The table highlights not only the policies themselves but also the supporting roles of media campaigns, advocacy organizations, and legal actions. Together, these cases demonstrate the practical relevance of the lifecycle approach, showing how different stakeholders and strategies have contributed at each phase of the enterprise.

#### 3.2.1. Conception & Initiation (framing + early governance)

The global framing of tobacco control began with the World Health Organization’s Framework Convention on Tobacco Control (FCTC), which created a comprehensive vision for reducing the harms of tobacco use worldwide. By establishing shared principles, scope, and obligations, the FCTC provided a foundation that emphasized health protection, international cooperation, and accountability mechanisms. In the United States, the California Tobacco Control Program (CTCP) has been a leader in translating this vision into action. The CTCP has not only advanced public education and prevention but has also explicitly backed local policies that move toward the elimination of commercial tobacco sales. Its commitment to equity and community involvement has anchored the program’s framing, making California a model for early governance and alignment of endgame goals.

#### 3.2.2. Policy & System Design (laws, rules, model ordinances)

Once the broad vision was set, the policy and system design phase involved codifying authority and establishing specific rules. A landmark example is the U.S. Family Smoking Prevention and Tobacco Control Act, which granted the Food and Drug Administration (FDA) regulatory authority over tobacco products and set restrictions on youth marketing. More recently, the FDA proposed product standards to prohibit menthol cigarettes and flavored cigars, publishing its notice in the *Federal Register* in May 2022. Although these rules have faced delays, they reflect how federal agencies design policies to target key drivers of inequities in tobacco use. States have also acted independently; Massachusetts passed a statewide flavored tobacco law in 2019, creating a model for other jurisdictions. At the national level, the Federal Tobacco-21 law raised the minimum purchase age to 21, with FDA providing compliance guidance for retailers. Meanwhile, California has relied on model ordinances—covering retail licensing, smoke- free environments, and sales restrictions—developed in partnership with organizations like the Public Health Law Center. These policies demonstrate how design frameworks support both legal authority and community adoption.

#### 3.2.3. Implementation & Operation (roll-outs, enforcement)

Policy design is only the beginning; effective implementation determines whether the intended outcomes are realized. The cities of Beverly Hills and Manhattan Beach in California became national leaders when they enacted citywide bans on tobacco sales, with Beverly Hills’ ordinance taking effect on January 1, 2021. Both cases illustrate how municipalities can operationalize end-of-sales while navigating exemptions, enforcement, and community response. Nationally, the FDA plays a central role in enforcement, issuing warning letters and civil money penalties to retailers and conducting compliance inspections. In recent years, the FDA has also partnered with the Department of Justice to create a multi-agency task force aimed at illicit e-cigarettes, showing how enforcement evolves to address emerging products. Together, these examples highlight how implementation requires coordination between local governments and federal regulators to ensure compliance and adapt to new market dynamics.

#### 3.2.4. Evaluation & Adaptation (monitoring, course-correction, courts)

Ongoing evaluation and adaptation are essential in a complex system. National surveillance systems such as the National Youth Tobacco Survey (NYTS) have documented major shifts in adolescent tobacco use, including historic lows in cigarette smoking reported in the 2024 *MMWR*. The Centers for Disease Control and Prevention (CDC) maintains regular updates on youth e-cigarette use, with its surveillance page most recently reviewed in March 2025. Oversight also comes from within government; the Office of Inspector General (OIG) at HHS issued a report in March 2025 recommending stronger penalties against repeat violators, prompting FDA to escalate its enforcement strategy. At the same time, courts play a critical role in forcing policy adaptation, as shown by a January 2025 federal court decision that blocked the FDA’s attempt to implement graphic warning labels. These examples show how evaluation mechanisms—whether epidemiological, administrative, or judicial—can reshape strategies and ensure responsiveness to evolving risks and challenges.

#### 3.2.5. Consolidation & Endgame Transition (integration toward sales elimination)

As policies and interventions accumulate, consolidation becomes necessary to transition toward an endgame framework. In California, local sales bans are scaling through the support of the CTCP and legal guidance from the Public Health Law Center, demonstrating how multiple ordinances can be integrated into a broader endgame pathway. International comparators also provide lessons: the United Kingdom’s *Tobacco and Vapes Bill* proposes a “smokefree generation” by gradually raising the legal purchase age, alongside a ban on disposable vapes starting in 2025. New Zealand had previously passed one of the most ambitious endgame laws—limiting sales to very low-nicotine cigarettes and reducing the number of retailers—before repealing it in 2024 due to political shifts. These examples underscore that consolidation and transition require not only legal and institutional integration but also sustained political will, which can be fragile and contested.

#### 3.2.6. Sustainment or Sunset (post-endgame maintenance)

Once the endgame is reached, maintaining gains becomes the challenge. Beverly Hills designed its ordinance with exemptions and scheduled reviews to ensure flexibility and adaptation over time, offering an early example of how post-endgame governance might operate. Manhattan Beach has sustained its earlier smoke-free outdoor places policies, reinforcing new norms long after initial adoption. These cases suggest that sustainment requires institutionalization of policies and cultural change, preventing regression and ensuring that the decline in tobacco use remains durable. The sunset phase may also involve redirecting resources to other public health priorities while keeping infrastructure in place to monitor and prevent relapse.

#### 3.2.7. Media & Communication (supporting the enterprise)

Throughout these phases, communication campaigns have been critical in reinforcing policy goals and shifting norms. The CDC’s *Tips From Former Smokers*^®^ campaign, last updated in 2025, has generated over one million sustained quits and more than 16 million quit attempts across multiple waves, including targeted ads focused on menthol. The Truth Initiative has complemented these efforts by developing innovative approaches, such as a randomized controlled trial–tested text messaging program to help teens quit vaping, published in *JAMA* in 2024. Campaigns have also increasingly addressed mental health linkages, broadening their relevance to youth audiences. These communication strategies illustrate how media can amplify policy implementation, personalize risks, and maintain public engagement across the lifecycle.

#### 3.2.8. Organizations & Associations (governance, advocacy, alignment)

Finally, professional associations and advocacy groups provide governance support and policy alignment. The American Medical Association (AMA) has issued repeated statements urging swift action on menthol restrictions, while the National Medical Association (NMA) has highlighted menthol as a racial equity issue for Black communities. The American Lung Association and the National Association of County and City Health Officials (NACCHO) have applied pressure through public statements and coalition letters to advance regulation. Legal expertise is provided by the Public Health Law Center, which maintains timelines, analyses, and litigation trackers, including cases like *AATCLC v. HHS*. At the same time, watchdog groups such as TobaccoTactics have documented industry countermeasures and delay strategies, reminding stakeholders of the need for vigilance. Collectively, these organizations help align policy goals with equity considerations, public support, and accountability structures.

#### 3.2.9. Recent Policy Volatility (signals of a complex system)

Recent events also remind us that tobacco control is a dynamic and contested system. The FDA’s menthol and flavored-cigar rules, first proposed in 2022, were formally withdrawn in January 2025 after significant delays, leaving states and localities to fill the gap. At the same time, enforcement capacity has been in flux, with large-scale staffing shifts within HHS and FDA reported, and the creation of a DOJ-FDA task force to address illicit e-cigarettes. These developments illustrate both the fragility and resilience of the system: while setbacks can delay progress, adaptive governance and local innovation continue to drive the enterprise forward.

### 3.3. Social Media and Stakeholder Communications Across the Lifecycle

Table 6 summarizes how social media platforms, government websites, municipal authorities, housing agencies, restaurant chains, and professional organizations have all contributed to different phases of the tobacco control lifecycle. These examples illustrate the breadth of actors that participate in shaping the U.S. tobacco endgame, from grassroots communication to federal oversight.

Social media platforms such as X (formerly Twitter) and Facebook have been instrumental in the conception, implementation, and sustainment phases. For instance, the CDC’s Tobacco Free account regularly posts *Tips From Former Smokers*^®^ content, amplifying quit stories and linking to resources, while the Campaign for Tobacco-Free Kids uses social media to frame youth tobacco prevention as a national priority. These posts not only reinforce public norms but also mobilize advocates, particularly around menthol regulation. Local action has also been broadcast on social media; for example, ABC10News posted about Beverly Hills’ historic retail sales ban on Facebook, signaling community-level norm change to both residents and visitors.

At the federal level, communication channels of Congress and agencies demonstrate the evaluation and adaptation phases. The House Energy & Commerce Subcommittee uses its website to announce hearings that scrutinize FDA’s tobacco programs, ensuring formal oversight. Similarly, the FDA’s own communications around enforcement, including its collaboration with the Department of Justice to form a multi-agency task force on illicit e-cigarettes, illustrate how operational updates and enforcement priorities are publicly shared and adapted to new risks.

California provides further evidence of early framing and policy design through the Department of Public Health and the California Tobacco Control Program, whose websites lay out an explicit vision of ending the commercial tobacco epidemic with a focus on health equity. State-level legislation, such as SB 793 and the voter-approved Proposition 31, was supported by public-facing resources that made the policy design process transparent. Local governments like Beverly Hills and Manhattan Beach also provide detailed implementation and compliance guidance through city websites, showing how municipalities use digital tools to operationalize bans and sustain local change.

Other sectors have also participated in implementation and sustainment. Public housing authorities, such as the Housing Authority of the City of Los Angeles, maintain smoke-free housing policies and resident education materials online, extending tobacco control to residential environments. Restaurant chains have long played a role in norm change: Starbucks’ outdoor smoking ban and McDonald’s nationwide smoke-free policy in the 1990s demonstrated how private-sector actions reinforce broader public health goals, and these corporate decisions continue to circulate through company websites and media reports.

Professional associations and advocacy organizations amplify these efforts during the consolidation phase. The American Medical Association and the National Medical Association have both issued policy statements on menthol regulation, frequently disseminated on their websites and social feeds. The American Lung Association and the National Association of County and City Health Officials use press releases and coalition letters to press for timely implementation of FDA rules, while the Public Health Law Center publishes litigation trackers to keep the public informed about industry counter-maneuvers. Advocacy groups such as TobaccoTactics document strategies used by the tobacco industry to delay or weaken policy, providing watchdog accountability.

Taken together, these examples demonstrate that the tobacco endgame is not confined to legislation or regulation alone. Instead, it operates as a multifaceted enterprise where communication platforms, organizational websites, and social media posts reinforce and extend each phase of the lifecycle. From conception to sustainment, digital and organizational communication channels serve to frame public discourse, mobilize constituencies, enforce compliance, and consolidate gains. These diverse modes of communication highlight how the endgame strategy depends on coordinated actions across sectors, ensuring resilience in the face of evolving industry tactics and changing political conditions.

To complement the conceptual framework, we identified phase-specific transition criteria, key performance indicators (KPIs), and equity safeguards that could guide decision-making across the lifecycle. Table 7 translates the six lifecycle phases into measurable thresholds, offering practical markers for when the enterprise should progress from one stage to the next. By linking these criteria to publicly available data sources, the framework provides accountability and transparency for both policymakers and advocates. Importantly, each phase includes an explicit equity check to ensure that policies and programs narrow, rather than widen, existing disparities in tobacco use and cessation. Such integration of disparities-sensitive measures reflects the need to align endgame efforts with the broader goal of health equity. By operationalizing transitions and embedding equity, this approach transforms the lifecycle model into a usable roadmap for practice and policy. Together, these criteria highlight how a structured enterprise framework can be both measurable and responsive to inequities in tobacco control.

### 3.4. Risk and Countermeasure Matrix Across the Lifecycle

Complex systems are inherently vulnerable to disruption, and tobacco control is no exception. Tabe 8 presents a structured risk and countermeasure matrix, highlighting foreseeable threats across the six lifecycle phases. These risks include legal challenges, industry innovation, enforcement gaps, misinformation campaigns, equity backfires, supply chain leakage, and political volatility. For each category, we identified leading indicators, the phases most likely to be affected, and practical mitigation strategies with responsible actors. This enterprise-style “risk register” underscores the importance of anticipatory governance and proactive countermeasures. Embedding such a matrix into the lifecycle model helps ensure resilience by preparing for setbacks rather than simply reacting to them. By naming responsible actors, the framework also clarifies accountability, strengthening the system’s capacity to adapt and endure.

### 3.5. International Comparators for Endgame Policies

Although tobacco control policies in the United States remain fragmented, valuable lessons can be drawn from international endgame efforts. Table 9 provides a comparative snapshot of endgame measures in the United Kingdom, New Zealand, Australia, and Canada, alongside current U.S. policies. Each country has pursued different approaches, including generational bans, nicotine reduction, retail density limits, plain packaging, and flavored product restrictions. These cases illustrate both the ambition and the fragility of endgame policies, as shown by New Zealand’s repeal of its bold legislation in 2024. By situating U.S. strategies within this global context, we can better understand the opportunities and risks of pursuing aggressive endgame measures. The table also highlights how different political, cultural, and regulatory environments influence the durability of reforms. Drawing on these examples may help U.S. policymakers design endgame strategies that are both innovative and sustainable.

## 4. Discussion

Complexity is managed through layering and abstraction [32,33], generating views and viewpoints into an object or system or enterprise. One method to create a set of views and viewpoints into the enterprises is Zachman Architecture Framework (ZAF) [34]. ZAF is a structured, conceptual schema for organizing and describing the architecture of complex systems—originally developed for information systems, but now applied more broadly to enterprises, engineering systems, and public policy [35,36]. It was created by John Zachman in the 1980s as a way to handle the complexity of large-scale systems by breaking them down into consistent perspectives and fundamental questions [34]. The core idea of ZAF is to generate a matrix that maps six interrogatives (What, How, Where, Who, When, Why) to some stakeholder perspectives related to the lifecycle of the system/enterprise under study. The intersections of the two views generate viewpoints that are easier to manage and understand individually, thus enabling complexity management.

Large-scale public health goals, particularly those targeting entrenched, adaptive challenges such as tobacco use, benefit from structured, enterprise-level planning. In management and systems engineering disciplines, enterprise refers to an organized, goal-directed system encompassing diverse stakeholders, processes, and resources working toward a shared mission [17]. When combined with a lifecycle perspective—breaking the enterprise into predictable, interdependent stages—this approach enables long-term planning, strategic alignment, and built-in adaptability.

The tobacco control movement in the United States, while historically successful in reducing smoking prevalence, has operated as a loose network of policies, programs, and advocacy campaigns. These have often been implemented reactively, in response to industry innovations or public health crises [3–9], rather than through a coordinated, phased enterprise strategy. The result has been substantial progress, yet with uneven outcomes across populations [37–39] and vulnerabilities to new product classes such as e-cigarettes and heated tobacco devices [40].

A lifecycle approach reframes tobacco control as an evolving national enterprise with a defined trajectory from inception to a sustained post-endgame environment. Each phase—whether initiating foundational research and coalition building, designing and implementing systems, adapting policies in response to emerging challenges, or managing the transition to the endgame—can be managed with enterprise-level governance, performance metrics, and cross-sectoral integration. This methodology ensures that efforts are not only effective in the short term but also resilient over decades, preventing regression and maintaining public health gains.

We provided the Tobacco Control Enterprise Lifecycle Model which provides a complexity management framework for orchestrating the multi-decade, multi-sector effort required to achieve the tobacco endgame. By structuring tobacco control as an evolving enterprise mapped through the Zachman Architecture Framework (ZAF), the model enables systematic identification of interdependencies, feedback loops, and systemic vulnerabilities. This complexity-aware perspective ensures that interventions are introduced and reinforced in a logical, sequenced manner—building foundational capacity before scaling ambitious endgame policies. Such sequencing mitigates the risks of legislation outpacing operational readiness or enforcement mechanisms lagging behind policy changes.

A core strength of the model lies in its capacity to integrate diverse sectors and disciplines within a coherent enterprise architecture. Tobacco control extends well beyond the remit of public health agencies; it requires the coordinated engagement of legislators, law enforcement, educators, healthcare providers, community leaders, and technology platforms that influence public discourse and marketing channels [41–45]. The Policy & System Design phase provides a structured governance framework to align these actors, while the Implementation & Operation phase focuses on synchronizing service delivery, enforcement actions, and public education campaigns.

To ensure sustained effectiveness in a volatile and adaptive landscape, the model incorporates process maturity assessment based on the Capability Maturity Model Integration (CMMI) [29]. This allows stakeholders to evaluate whether processes at each lifecycle phase are ad hoc (Level 1), repeatable (Level 2), standardized (Level 3), quantitatively managed (Level 4), or optimized for continuous improvement (Level 5). By embedding maturity assessment, the framework enables targeted capability building, ensuring that enterprise functions advance in sophistication in parallel with policy ambition.

Table 4 illustrated how measurable indicators can be applied to monitor performance across the Zachman dimensions in real time. For example, in the Conception & Initiation phase, metrics such as the percentage of states with current prevalence data and the number of policy concept papers produced provide a tangible assessment of foundational readiness. In Implementation & Operation, measures such as percentage of planned campaigns delivered on schedule and compliance inspection coverage serve as operational performance benchmarks. In later phases, such as Consolidation & Endgame Transition, indicators like endgame law enactment rates, cross-border enforcement coverage, and synchronization of final measures nationally offer an evidence base for gauging readiness to close out the enterprise.

The results of our structured search underscore that the tobacco endgame is already being tested in practice, both in the United States and abroad. Table 5 illustrated how interventions can be mapped across six lifecycle phases, ranging from conception and initiation through sustainment or sunset. These real-world examples demonstrate that the lifecycle model is not simply a theoretical framework but a practical lens for understanding how disparate actions interact across governance levels. For example, the Family Smoking Prevention and Tobacco Control Act represents a pivotal moment of policy design, while Beverly Hills’ retail sales ban illustrates municipal-level implementation. Equally important are cross-cutting elements such as communication campaigns and advocacy organizations, which act as connective tissue linking otherwise siloed policy actions. Collectively, these cases highlight how endgame efforts require coordination across multiple layers of the system and depend on both top-down regulation and bottom-up mobilization.

Building on this, Table 6 showed how communication channels—including social media, organizational websites, and professional associations—reinforce lifecycle phases in real time. The use of platforms such as Twitter (X), Facebook, YouTube, and TikTok illustrates how digital communication serves as both a monitoring tool and a vehicle for public mobilization. For instance, CDC’s Tips From Former Smokers^®^ campaign maintains visibility and reach in the sustainment phase, while activist-led TikTok challenges engage youth directly during the implementation and consolidation phases. Municipalities and state agencies also use digital portals to publicize compliance guidance, signaling how governance itself is increasingly mediated through online channels. These findings reinforce that the enterprise of tobacco control is not only enacted through legislation but also amplified and normalized through communication infrastructures that shape norms and expectations.

To ensure that the lifecycle approach is actionable, Table 7 introduced measurable transition criteria, key performance indicators, and equity checks for each phase. These criteria provide a roadmap for when to move from one phase to the next, ensuring that policies advance only when core conditions are met. Importantly, the integration of explicit equity safeguards reflects the need to avoid widening disparities—a risk that has been well documented in other areas of public health. By embedding metrics such as parity in cessation service reach or reductions in menthol use disparities, the framework helps operationalize the commitment to health equity within the endgame itself. In doing so, it translates a conceptual model into a set of measurable and evaluable benchmarks that can guide federal, state, and local action.

At the same time, Table 8 acknowledged that a lifecycle approach must contend with risks and vulnerabilities inherent in complex adaptive systems. Legal challenges, industry innovation, enforcement gaps, misinformation, and political volatility are not outliers but expected features of a contested system. By framing these risks alongside countermeasures and responsible actors, the table functioned as a risk register for the tobacco endgame. This anticipatory approach reflects a key principle of complexity management: resilience comes not from eliminating shocks but from preparing to absorb and adapt to them. Embedding such a risk lens into the lifecycle model makes it more robust and realistic, ensuring that policy ambition is matched with operational foresight.

Finally, Table 9 situated U.S. tobacco control within an international context, comparing endgame measures in the United Kingdom, New Zealand, Australia, and Canada. These cases illustrated both the ambition of global endgame strategies and their vulnerability to political reversal, as seen in New Zealand’s repeal of its pioneering law. For U.S. stakeholders, these examples provide cautionary lessons about the fragility of political will and the need to build durable, equity-centered coalitions to sustain bold reforms. They also highlight the importance of sequencing and design: while some countries have pursued generational sales bans, others have relied on retail density limits, excise taxes, or flavor restrictions. This diversity of strategies underscores that there is no single path to the endgame but rather a portfolio of approaches that can be adapted to local contexts.

Taken together, the findings summarized in Tables 5–9 demonstrated that the lifecycle model is both empirically grounded and practically relevant. They showed how U.S. tobacco control has already moved through multiple phases, how communication and advocacy sustain momentum, how measurable thresholds can guide transitions, how risks can be anticipated and managed, and how international experiences can inform U.S. planning. This integration of theory, evidence, and practice strengthens the case for treating the tobacco endgame as a managed enterprise rather than a series of disconnected interventions.

Adaptability is another critical pillar of complexity management in this model. The Evaluation & Adaptation phase institutionalizes rapid feedback loops, ensuring that policy and operational strategies evolve in response to emerging products, market tactics, and shifting social norms. The U.S. experience with e-cigarettes—where uptake among youth outpaced regulatory adaptation [46,47]—underscores the necessity of such real-time responsiveness in complex systems.

The model’s final phases—Consolidation & Endgame Transition and Sustainment or Sunset—address the challenges of preserving system stability after achieving near-zero prevalence. This involves implementing bold, final measures such as nicotine content reduction, sales bans for future generations, and international supply chain controls, while also anticipating potential resurgence via illicit markets or new addictive products. Sustaining a post-tobacco culture requires not only ongoing education but also the preservation of institutional memory so that learned lessons, operational networks, and strategic capabilities are not lost over time.

Despite substantial progress in reducing cigarette smoking over the past five decades, tobacco use remains the leading cause of preventable death in the United States, causing over 480,000 deaths annually [48]. The tobacco epidemic’s complexity—shaped by industry innovation, socio-economic disparities, and the emergence of new nicotine delivery systems—demands a systemic, architecture-driven approach.

By doing so, the model transforms tobacco control from a series of reactive measures into a proactive, strategically sequenced, and continuously improving national enterprise. Its application has the potential to improve policy coherence, optimize resource allocation, and enhance resilience against both anticipated and unforeseen challenges—ultimately safeguarding the long-term public health gains envisioned by the tobacco endgame.

## 5. Conclusion

The Tobacco Control Enterprise Lifecycle Model offers a comprehensive, stage-based roadmap for achieving the tobacco endgame in the United States. By framing tobacco control as an evolving enterprise, it enables policymakers and public health leaders to plan strategically, align actions across sectors, and adapt to emerging challenges. Its sequential phases ensure that foundational work in capacity-building and policy design is solidified before moving toward transformative measures.

This approach not only supports progress toward elimination but also safeguards against regression, ensuring that public health gains are sustained in a post-tobacco era. As the U.S. confronts the dual challenges of persistent disparities and a diversifying nicotine market, adopting a lifecycle perspective can provide the clarity and coordination necessary to accelerate the endgame timeline.

## Figures and Tables

**Table 1.** Views and Viewpoints of the U.S. Tobacco Control Enterprise Using Zachman Interrogatives

Lifecycle Phase	What (Data / Elements / Products)	How (Processes / Functions)	Where (Locations / Networks)	Who (Stakeholders / Actors)	When (Timeframes / Events)	Why (Goals / Motivations)
1. Conception & Initiation	Epidemiological data, mortality statistics, cost-of-smoking studies, FCTC provisions, draft mission statement	Situational analysis, stakeholder mapping, advocacy planning	Federal policy circles (Congress, HHS), state health departments, public health summits	CDC Office on Smoking and Health, FDA CTP, Surgeon General, advocacy NGOs, tribal health leaders	Initial policy momentum periods, post-Surgeon General reports, pre-election cycles	Build mandate for tobacco control, align with WHO FCTC, secure funding and political commitment
2. Policy & System Design	Draft legislation, regulatory frameworks, taxation policy drafts, organizational charts, performance metric templates	Legislative drafting, regulatory impact analysis, organizational design, budget allocation	Capitol Hill, state legislatures, federal agencies, state DOHs	Federal lawmakers, state legislators, public health lawyers, economic analysts, advocacy coalitions	Legislative sessions, budget cycles, FCTC reporting deadlines	Create enforceable, evidence-based laws and structures for tobacco control
3. Implementation & Operation	Public campaign materials, Quitline infrastructure, NRT stock, inspection reports, compliance databases	Campaign deployment, cessation service delivery, retailer inspections, tax collection enforcement	Community health centers, schools, retail outlets, enforcement field offices	Public health educators, Quitline staff, enforcement officers, local NGOs	Annual public health campaign launches, quarterly compliance sweeps, continuous cessation service availability	Reduce smoking initiation, support quitting, ensure compliance with laws
4. Evaluation & Adaptation	Surveillance datasets, program evaluation reports, illicit trade detection data, vaping prevalence surveys	Data analysis, policy review, stakeholder feedback collection, regulatory adjustment	National data repositories, research institutions, policy workshops	Epidemiologists, academic researchers, policymakers, advocacy leaders	Annual CDC surveys (e.g., NHIS, YRBSS), biennial program evaluations	Improve program effectiveness, respond to emerging products and threats
5. Consolidation & Endgame Transition	Endgame policy packages (smokefree generation law, nicotine reduction regs, sinking lid supply quotas), cross-border enforcement agreements	Intensive cessation targeting, final supply reduction, global coordination	National enforcement hubs, customs checkpoints, international treaty meetings	Federal agencies (FDA, CBP), WHO FCTC Secretariat, neighboring country regulators	Final push period before prevalence <5%, synchronized international enforcement	Eliminate commercial tobacco use, prevent cross-border leakage
6. Sustainment / Sunset	Post-endgame monitoring systems, cultural norm education materials, illicit trade intelligence systems	Long-term surveillance, preventive education, resource reallocation to other public health areas	Local community health networks, schools, customs & border patrol	Sustained public health teams, community educators, law enforcement	Continuous post-endgame monitoring cycles	Prevent relapse, maintain tobacco-free norms, safeguard public health gains

**Table 2.** A quick refresher on CMMI Levels in a public health policy context

CMMI Level	Description	Public Health / Tobacco Control Analogy
1 – Initial	Unpredictable, reactive, ad hoc processes.	Sporadic or pilot initiatives without systematic enforcement.
2 – Managed	Projects are planned, documented, and tracked.	Structured campaigns, clear program ownership, repeatable at project level.
3 – Defined	Processes are standardized and integrated organization-wide.	Nationally standardized policy frameworks, coordinated across agencies.
4 – Quantitatively Managed	Processes are measured, monitored, and controlled with data.	Performance metrics and surveillance data actively drive program decisions.
5 – Optimizing	Continuous improvement based on quantitative feedback.	Dynamic adaptation of programs to emerging products/threats, endgame innovation.

**Table 3.** Measures & Variables – Zachman Cells for U.S. Tobacco Endgame

1. WHAT — Data / Elements / Products Goal: Assess completeness and quality of endgame-relevant artifacts (laws, datasets, infrastructure).	Conception & Initiation	Availability of baseline prevalence & mortality datasets; Endgame policy concept papers produced	% of states with current prevalence data; # of policy concept papers published
	Policy & System Design	Completeness of draft legislation & regulatory frameworks	% of FCTC-recommended measures included; # of policy gaps identified
	Implementation & Operation	Campaign material coverage; Quitline/NRT resource stock	% of population reached by campaigns; NRT supply adequacy index
	Evaluation & Adaptation	Timeliness and completeness of surveillance datasets	Avg. lag (months) between data collection and release; % of data fields complete
	Consolidation & Endgame Transition	Endgame law enactment rate; Supply reduction instruments in place	% of endgame measures legislated; # of retail outlets under supply quotas
	Sustainment / Sunset	Post-endgame monitoring systems operational	% of illicit trade cases detected vs prosecuted; # of public norm reinforcement materials issued per year
2. HOW — Processes / Functions Goal: Evaluate maturity and consistency of endgame-related processes.	Conception & Initiation	Quality of stakeholder engagement processes	# of stakeholder meetings; % of priority stakeholders engaged
	Policy & System Design	Process adherence to regulatory impact assessment standards	% of policy drafts with documented impact analysis; review cycle time (days)
	Implementation & Operation	Campaign deployment effectiveness; Compliance inspection coverage	% of planned campaigns delivered; # of inspections per 1,000 retailers
	Evaluation & Adaptation	Responsiveness of policy adjustments	Avg. time from new threat detection to policy update
	Consolidation & Endgame Transition	Execution speed of final cessation pushes	% of remaining smokers reached with targeted support; weeks to implement supply quota
	Sustainment / Sunset	Continuity of surveillance and education	% of planned monitoring events completed; % of educational programs renewed annually
3. WHERE — Locations / Networks Goal: Measure geographic reach and network integration.	Conception & Initiation	Geographic coverage of baseline data	% of states with reliable baseline prevalence measures
	Policy & System Design	State-level alignment with federal frameworks	% of states with harmonized endgame policies
	Implementation & Operation	Geographic reach of campaigns and enforcement	% of population in covered jurisdictions; inspections per region
	Evaluation & Adaptation	Distribution of data collection sites	% of counties with active surveillance points
	Consolidation & Endgame Transition	Cross-border enforcement coverage	# of customs checkpoints integrated into enforcement plan
	Sustainment / Sunset	Post-endgame coverage of illicit trade monitoring	% of high-risk border zones under continuous monitoring
4. WHO — Stakeholders / Actors Goal: Assess breadth and alignment of actors.	Conception & Initiation	Diversity of engaged stakeholder groups	# of unique stakeholder categories (gov, NGO, research, community)
	Policy & System Design	Representation in policy design forums	% of policy design meetings with multi-sector participation
	Implementation & Operation	Stakeholder participation in campaign delivery	% of NGOs actively delivering campaign components
	Evaluation & Adaptation	Multi-stakeholder involvement in evaluation	% of evaluation panels with non-gov participants
	Consolidation & Endgame Transition	Cross-agency coordination effectiveness	# of joint operations conducted; % of joint objectives met
	Sustainment / Sunset	Community engagement in sustaining tobacco-free norms	% of community groups funded for prevention activities
5. WHEN — Timeframes / Events Goal: Evaluate timeliness and synchronization.	Conception & Initiation	Time from problem identification to initial strategy draft	Avg. months elapsed
	Policy & System Design	Policy drafting cycle time	Avg. days from drafting start to legislative submission
	Implementation & Operation	Campaign and inspection timeliness	% of activities delivered on schedule
	Evaluation & Adaptation	Speed of response to emerging threats	Avg. days to policy update after detection
	Consolidation & Endgame Transition	Synchronization of final measures nationally	% of states implementing endgame measures within target window
	Sustainment / Sunset	Frequency of post-endgame monitoring events	Avg. interval (months) between surveillance reports
6. WHY — Goals / Motivations Goal: Measure clarity, alignment, and adaptability of goals.	Conception & Initiation	Specificity of mission statements	% of mission statements with measurable targets
	Policy & System Design	Alignment of policy goals with national health targets	% of goals explicitly tied to Healthy People objectives or FCTC
	Implementation & Operation	Alignment of program KPIs to strategic goals	% of program KPIs mapping to national endgame targets
	Evaluation & Adaptation	Use of evaluation findings to adjust goals	% of updated goals following evaluation cycles
	Consolidation & Endgame Transition	Goal clarity in final push phase	% of endgame policies with specific prevalence targets
	Sustainment / Sunset	Maintenance of post-endgame vision	% of strategic plans including long-term relapse prevention objectives

**Table 4.** Zachman Cell Measures with Data Source Examples

Lifecycle Phase	Interrogative	Measures	Variables	Potential Data Sources / AI Search Examples
1. Conception & Initiation	What	Availability of baseline prevalence & mortality datasets; Endgame policy concept papers produced	% of states with current prevalence data; # of policy concept papers published	CDC NHIS, BRFSS, STATE System for prevalence; state health dept reports; Surgeon General reports
	How	Quality of stakeholder engagement processes	# of stakeholder meetings; % of priority stakeholders engaged	NAQC quitline annual survey; minutes from state tobacco control coalition meetings
	Where	Geographic coverage of baseline data	% of states with reliable baseline prevalence measures	CDC STATE maps; county-level BRFSS prevalence data
	Who	Diversity of engaged stakeholder groups	# of unique stakeholder categories (gov, NGO, research, community)	NAQC survey partner lists; state tobacco advisory board rosters
	When	Time from problem identification to initial strategy draft	Avg. months elapsed	Legislative records; news archives; HHS press releases
	Why	Specificity of mission statements	% of mission statements with measurable targets	State tobacco control plans; CDC OSH national strategy documents
2. Policy & System Design	What	Completeness of draft legislation & regulatory frameworks	% of FCTC-recommended measures included; # of policy gaps identified	CDC **STATE System** policy coverage; state legislative tracking (NCSL)
	How	Process adherence to regulatory impact assessment standards	% of policy drafts with documented impact analysis; review cycle time (days)	Federal Register entries; GAO reports on tobacco regulation
	Where	State-level alignment with federal frameworks	% of states with harmonized endgame policies	CDC STATE policy alignment datasets
	Who	Representation in policy design forums	% of policy design meetings with multi-sector participation	Legislative hearing transcripts; public comment registries
	When	Policy drafting cycle time	Avg. days from drafting start to legislative submission	Bill histories from Congress.gov and state legislature portals
	Why	Alignment of policy goals with national health targets	% of goals tied to Healthy People or FCTC objectives	State tobacco plans vs Healthy People TU objectives
3. Implementation & Operation	What	Campaign material coverage; Quitline/NRT resource stock	% of population reached by campaigns; NRT supply adequacy index	CampaignReach metrics; NAQC Quitline reports; NRT distribution records
	How	Campaign deployment effectiveness; Compliance inspection coverage	% of planned campaigns delivered; # of inspections per 1,000 retailers	FDA Tobacco Compliance Check database; campaign progress reports
	Where	Geographic reach of campaigns and enforcement	% of population in covered jurisdictions; inspections per region	FDA inspection location data + Census population maps
	Who	Stakeholder participation in campaign delivery	% of NGOs actively delivering campaign components	NAQC program participation data
	When	Campaign and inspection timeliness	% of activities delivered on schedule	Campaign schedules; FDA enforcement calendar
	Why	Alignment of program KPIs to strategic goals	% of program KPIs mapping to national endgame targets	State DOH performance plans; CDC OSH performance dashboards
4. Evaluation & Adaptation	What	Timeliness and completeness of surveillance datasets	Avg. lag (months) between data collection and release; % of data fields complete	CDC YRBSS, NYTS release calendars; STATE System update logs
	How	Responsiveness of policy adjustments	Avg. time from new threat detection to policy update	FDA guidance issuance timelines; policy change notices
	Where	Distribution of data collection sites	% of counties with active surveillance points	YRBSS site list; state survey sampling frames
	Who	Multi-stakeholder involvement in evaluation	% of evaluation panels with non-gov participants	Panel rosters; evaluation meeting minutes
	When	Speed of response to emerging threats	Avg. days to policy update after detection	DOJ/FDA illicit trade enforcement announcements
	Why	Use of evaluation findings to adjust goals	% of updated goals following evaluation cycles	Revision histories in state tobacco control plans
5. Consolidation & Endgame Transition	What	Endgame law enactment rate; Supply reduction instruments in place	% of endgame measures legislated; # of retail outlets under supply quotas	CDC policy tracking; state licensing data
	How	Execution speed of final cessation pushes	% of remaining smokers reached with targeted support; weeks to implement supply quota	State cessation program reports; NRT procurement records
	Where	Cross-border enforcement coverage	# of customs checkpoints integrated into enforcement plan	CBP operation logs; DOJ task force reports
	Who	Cross-agency coordination effectiveness	# of joint operations conducted; % of joint objectives met	MOUs; joint task force meeting reports
	When	Synchronization of final measures nationally	% of states implementing endgame measures within target window	Policy adoption timelines from CDC STATE
	Why	Goal clarity in final push phase	% of endgame policies with specific prevalence targets	Legislative text review; agency strategic plans
6. Sustainment / Sunset	What	Post-endgame monitoring systems operational	% of illicit trade cases detected vs prosecuted; # of norm reinforcement materials issued/year	CBP/DOJ enforcement stats; public health education archives
	How	Continuity of surveillance and education	% of planned monitoring events completed; % of educational programs renewed annually	Surveillance schedules; funding renewal records
	Where	Post-endgame coverage of illicit trade monitoring	% of high-risk border zones under continuous monitoring	CBP surveillance maps; enforcement jurisdiction reports
	Who	Community engagement in sustaining tobacco-free norms	% of community groups funded for prevention activities	Grant award databases; program rosters
	When	Frequency of post-endgame monitoring events	Avg. interval (months) between surveillance reports	Report publication dates
	Why	Maintenance of post-endgame vision	% of strategic plans including relapse prevention objectives	Strategic plan review; public health policy archives

**Table 5.** Real-World Examples of U.S. Tobacco Control Across Lifecycle Phases

Phase	Illustrative Examples	Key Evidence / Outcomes
1. Conception & Initiation	WHO FCTC framing; California Tobacco Control Program (CTCP) vision	Established governance, equity framing, and end-of-sales pathways
2. Policy & System Design	Family Smoking Prevention and Tobacco Control Act; FDA proposed menthol & flavored cigar rules; Massachusetts statewide flavor ban; Federal Tobacco-21 law; CA model ordinances	Legal authority, product standards, age restrictions, and design templates for localities
3. Implementation & Operation	Beverly Hills and Manhattan Beach retail sales bans; FDA retail enforcement and illicit-vape task force	Roll-out of local end-of-sales; federal compliance checks and penalties
4. Evaluation & Adaptation	National Youth Tobacco Survey (2024) MMWR; CDC e-cigarette updates; HHS OIG enforcement review; litigation blocking FDA graphic warnings	Surveillance informs adaptation; oversight strengthens penalties; courts reshape strategies
5. Consolidation & Endgame Transition	Local end-of-sales scaling via CTCP; international comparators: UK Smokefree Generation bill, NZ endgame law (later repealed)	Integration of policies, cross-sector alignment, and global learning
6. Sustainment or Sunset	Beverly Hills ordinance with exemptions and reviews; Manhattan Beach smoke-free outdoor policies	Institutionalized governance, norm maintenance, and long-term stability
Cross-cutting: Media & Communication	CDC “Tips From Former Smokers^®^”; Truth Initiative quit-vaping programs; campaign RCTs in *JAMA*	National reach campaigns, >1M sustained quits, teen text programs with measurable quit rates
Cross-cutting: Organizations & Associations	AMA, NMA, ALA, NACCHO advocacy; Public Health Law Center litigation tracker; TobaccoTactics documentation	Professional societies and advocacy groups frame equity, apply pressure, and track industry counter-measures

**Table 6.** Social media and stakeholder communications mapped to the six-phase lifecycle

Channel / sector	Entity (example)	What it shows	Lifecycle phase(s)
X / Twitter	CDC Tobacco Free	Tips^®^ campaign posts highlighting quit stories and linking to resources—norm shaping and cessation support amplified via social.	3 Implementation; 4 Evaluation; 6 Sustainment
X / Twitter	Campaign for Tobacco-Free Kids	Advocacy content around youth prevention and support for proven campaigns—agenda setting and public mobilization.	1 Conception; 5 Consolidation
Facebook (media pickup of municipal action)	ABC10News post on Beverly Hills	Wide public dissemination of the Beverly Hills retail sales ban; signals local norm change to residents and visitors.	3 Implementation; 6 Sustainment
U.S. Congress (official site)	House Energy & Commerce Subcommittee hearing page	Congressional oversight hearing on FDA human foods and tobacco programs—formal evaluation/feedback loop.	4 Evaluation & adaptation
California state (program site)	CDPH / California Tobacco Control Program (CTCP)	State vision and objectives that explicitly build capacity to “end the commercial tobacco epidemic,” with equity focus.	1 Conception; 5 Consolidation
State legislation (California)	SB 793 (flavored tobacco sales ban) + Prop 31 (2022 referendum upholding it)	Statutory design + public ratification of endgame-aligned sales restrictions.	2 Policy & system design; 5 Consolidation
Municipal (ordinance info)	City of Beverly Hills business guidance	Clear implementation details and compliance guidance for the citywide retail sales ban (effective Jan 1, 2021).	3 Implementation; 6 Sustainment
Municipal (adoption record)	Manhattan Beach Legistar docket & city page	Formal introduction and adoption of a comprehensive retail sales ban; complementary smoke-free public places.	3 Implementation
Federal enforcement (news + agency)	DOJ–FDA multi-agency task force; FDA CTP enforcement hub	Coordinated crackdown on illegal e-cigs; warnings/CMPs; public dashboards—operational response to emergent risks.	3 Implementation; 4 Evaluation
Organizations (medical)	AMA statements urging menthol action	Physician advocacy pressing for timely finalization of menthol/flavored cigar rules; equity framing.	5 Consolidation
Organizations (Black physicians)	National Medical Association statements/resources	Equity-oriented support for menthol prohibition and stronger enforcement.	5 Consolidation
Organizations (public health NGOs)	American Lung Association press + SoTC 2025	Public updates on rule delays; annual grading of state/federal policies; framing industry interference.	4 Evaluation; 5 Consolidation
Housing (public)	HACLA Smoke-Free Policy & resident materials	Comprehensive smoke-free rules across housing authority properties—policy diffusion beyond retail.	3 Implementation; 6 Sustainment
Restaurants (corporate policies)	Starbucks outdoor smoking ban (2013)	Corporate policy eliminating smoking within 25 ft of stores—norm reinforcement in hospitality settings.	3 Implementation; 6 Sustainment
Restaurants (corporate policies)	McDonald’s smoke-free move (1994)	Early, high-visibility corporate shift to smoke-free restaurants nationwide; accelerates social norm change.	3 Implementation; 6 Sustainment
YouTube	“Eliminating Commercial Tobacco: The Endgame Approach” video	Public health–oriented educational content on endgame strategies	1 Conception; 2 Policy design
YouTube	CTCP (California Tobacco Control Program) playlist	State-level mission videos aligned with tobacco control vision	1 Conception; 5 Consolidation
TikTok	#ImmuneUpVapesDown challenge (Truth Initiative)	Youth-engaged anti-vaping campaign via influencers	3 Implementation; 5 Consolidation
TikTok / media	Study on message framing in TikTok videos	Research on how TikTok messaging affects youth intention to quit vaping	4 Evaluation & Adaptation
Activists	Social media influencers combating youth vaping	Teens using Instagram/TikTok to create anti-vape messaging	1 Conception; 3 Implementation
School websites	K–12 district policy PDFs and policy builders	Model policies and tools for creating tobacco-free school districts	2 Policy design; 3 Implementation
Universities	UC system and CSU campuses smoke/tobacco-free policies	Comprehensive campus-wide restrictions on tobacco use	3 Implementation; 6 Sustainment
University websites	UCLA Healthy Campus Initiative	Integrated health promotion, including tobacco-free norms	1 Conception; 6 Sustainment
Doctors / Hospitals	Naval Hospital Camp Pendleton tobacco cessation program	Direct smoking/vaping cessation services for military personnel	3 Implementation; 6 Sustainment
Doctors / Hospitals	Rush Medical Center smoking cessation services	Evidence-based coaching, medications, QuitLine links	3 Implementation; 4 Evaluation
Research centers	Johns Hopkins Institute for Global Tobacco Control lectures (YouTube)	Knowledge dissemination and idea-sharing among experts	4 Evaluation; 5 Consolidation
Tobacco Industry	(Not captured in search results yet)	Industry communication platforms promoting novel nicotine products	4 Evaluation; 5 Consolidation (noted threat)

**Table 7.** Phase-Specific Transition Criteria, KPIs, and Equity Checks

Phase → Next Phase	Core Transition Criteria	Key Performance Indicators (KPIs)	Equity Check (Disparities-Sensitive)	Illustrative Data Sources
1 Conception → 2 Design	≥48 states with current prevalence baselines; ≥80% of priority stakeholders engaged	# stakeholder sessions; % mission statements with measurable targets	Equity objectives explicitly included (e.g., menthol, targeted marketing)	CDC NHIS; BRFSS; YRBSS/NYTS; STATE System; state tobacco plans
2 Design → 3 Implementation	≥75% of drafted bills include impact analysis; ≥60% of jurisdictions aligned with federal/state frameworks	Policy drafting cycle time; # of model ordinances adopted	Equity impact assessments completed; anti-preemption safeguards documented	Federal Register; NCSL; state legislature portals
3 Implementation → 4 Evaluation	≥85% of planned campaigns launched; ≥70% retailer inspection coverage; Quitline capacity ≥95%	Campaign reach (% of population); inspections per 1,000 retailers; NRT adequacy index	Service reach parity within 10% across racial/SES groups	FDA compliance database; NAQC reports; state dashboards
4 Evaluation → 5 Consolidation	<8% adult prevalence and ≥20% year-over-year decline in youth vaping	Data release lag <6 months; policy update latency <120 days	Reduction in menthol use disparities ≥25%	MMWR/NYTS; OIG/FDA enforcement logs
5 Consolidation → 6 Sustainment	Endgame package enacted in ≥50% of states; cross-border enforcement coverage ≥80%	% retailers under supply controls; % targeted cessation coverage	No widening of disparities during final push	STATE System; CBP/DOJ taskforce reports
6 Sustainment (steady-state)	Illicit trade cases resolved ≥70%; relapse rate stable/declining over 3 years	Monitoring frequency; community education renewal rate	Prevention grants distributed proportional to burden	CBP/DOJ; state prevention grants

**Table 8.** Risk and Countermeasure Matrix Across the Lifecycle

Risk Category	Leading Indicators / Triggers	Likely Impacted Phases
Litigation & Preemption	Court injunctions; state preemption bills introduced	2 Design; 4 Evaluation; 5 Consolidation
Industry Product Pivot	Surge in disposables or heated products; flavor substitutions	3 Implementation; 4 Evaluation
Enforcement Gaps	Inspection backlogs; weak penalties for repeat violators	3 Implementation; 4 Evaluation
Misinformation / Social Media	Viral pro-vape content; youth influencer marketing	1 Conception; 3 Implementation; 6 Sustainment
Equity Backfire	Cessation access gaps; menthol substitution	3 Implementation; 5 Consolidation
Supply Chain Leakage	Cross-border sales; illicit markets growth	5 Consolidation; 6 Sustainment
Political Volatility	Policy repeals; leadership turnover	1 Conception; 5 Consolidation

**Table 9.** International Comparators for Endgame Policies

Country	Core Endgame Measures	Implementation Status	Relevance for U.S.
United Kingdom	Smokefree Generation law (progressively raising age of sale); disposable vape ban (2025)	Bill under debate in Parliament; implementation timelines announced	Provides template for gradual endgame measures with youth focus
New Zealand	Reduced nicotine content mandate; retail density reduction; generational sales ban	Enacted 2022; repealed 2024 due to political shifts	Shows both ambition and fragility of bold endgame measures
Australia	Prescription-only nicotine vaping products; plain packaging; high excise taxes	Active enforcement; ongoing evaluation	Demonstrates strong federal regulation and supply control
Canada	National menthol ban; provincial retail restrictions; plain packaging	Fully implemented; evaluation data available	Illustrates equity-driven regulation targeting flavored products
United States (current)	Tobacco-21 law; state/local flavored tobacco bans; FDA proposed menthol rule (withdrawn 2025)	Patchwork adoption; uneven enforcement	Highlights need for coordinated national endgame strategy

## Abbreviations

**Table:**

Abbreviation	Full Term	Brief Definition
AATCLC	African American Tobacco Control Leadership Council	Advocacy group; litigant in tobacco control cases (e.g., vs. HHS).
ALA	American Lung Association	National nonprofit focused on lung health and tobacco control.
AMA	American Medical Association	U.S. physicians’ association; issues tobacco policy statements.
BRFSS	Behavioral Risk Factor Surveillance System	CDC state-based adult health survey.
CBP	U.S. Customs and Border Protection	Federal agency; cross-border enforcement (e.g., illicit trade).
CDC	Centers for Disease Control and Prevention	U.S. public health agency (surveillance, guidance).
CDU	Charles R. Drew University of Medicine and Science	Institutional affiliation of authors.
CMMI	Capability Maturity Model Integration	Process-maturity framework adapted here for public health systems.
CTP	Center for Tobacco Products (FDA)	FDA office regulating tobacco products.
CTCP	California Tobacco Control Program	California’s state tobacco control program.
DOJ	U.S. Department of Justice	Federal law enforcement; partners with FDA on illicit e-cigs.
DOH / DPH	Department of Health / Department of Public Health	State/local health agencies.
EU	European Union	Supranational entity; sometimes used for comparative policy context.
FCTC	Framework Convention on Tobacco Control	WHO treaty guiding global tobacco control.
FDA	U.S. Food and Drug Administration	Federal regulator of tobacco products (via CTP).
GAO	Government Accountability Office	U.S. congressional watchdog; evaluates federal programs.
HHS	U.S. Department of Health and Human Services	Federal department housing FDA, CDC, OIG, etc.
IGTC	(Johns Hopkins) Institute for Global Tobacco Control	Research center disseminating tobacco control evidence.
JAMA	Journal of the American Medical Association	Medical journal (e.g., RCTs on quit-vaping programs).
KPI	Key Performance Indicator	Quantitative metric to track program goals.
MMWR	Morbidity and Mortality Weekly Report	CDC publication for surveillance findings.
NACCHO	National Association of County and City Health Officials	Local public health association; policy/advocacy.
NMA	National Medical Association	Association of Black physicians; equity-focused tobacco policy.
NHIS	National Health Interview Survey	CDC national health survey of U.S. adults.
NRT	Nicotine Replacement Therapy	Pharmacotherapy for smoking cessation.
NYTS	National Youth Tobacco Survey	CDC/FDA national survey of youth tobacco use.
OIG	Office of Inspector General (HHS)	Oversight body; evaluates enforcement and compliance.
OSH	Office on Smoking and Health (CDC)	CDC office leading tobacco prevention/control.
Prop 31	California Proposition 31 (2022)	Referendum upholding CA flavored tobacco sales ban (SB 793).
RCT	Randomized Controlled Trial	Study design evaluating intervention efficacy.
SB 793	California Senate Bill 793	State law prohibiting flavored tobacco sales.
SoTC	State of Tobacco Control (ALA)	Annual ALA report grading tobacco policies.
STATE System	State Tobacco Activities Tracking and Evaluation System	CDC policy tracking database.
TRL	Tobacco Retail License	Local/state licensing framework for retailers.
UCLA	University of California, Los Angeles	Example university site for campus health initiatives.
UniSA	University of South Australia	Institutional affiliation of author.
WHO	World Health Organization	UN specialized agency for public health.
YRBSS	Youth Risk Behavior Surveillance System	CDC survey of youth risk behaviors (including tobacco).
ZAF	Zachman Architecture Framework	Enterprise architecture framework used in this model.
